# Physical Health and Transition to Psychosis in People at Clinical High Risk

**DOI:** 10.3390/biomedicines12030523

**Published:** 2024-02-26

**Authors:** Andrea De Micheli, Umberto Provenzani, Kamil Krakowski, Dominic Oliver, Stefano Damiani, Natascia Brondino, Philip McGuire, Paolo Fusar-Poli

**Affiliations:** 1 Early Psychosis: Interventions and Clinical-Detection (EPIC) Lab, Department of Psychosis Studies, Institute of Psychiatry, Psychology & Neuroscience, King’s College London, London SE5 8AB, UK; andrea.de_micheli@kcl.ac.uk (A.D.M.); kamil.krakowski@kcl.ac.uk (K.K.); dominic.a.oliver@kcl.ac.uk (D.O.); 2 OASIS Service, South London and Maudsley NHS Foundation Trust, London SE11 5DL, UK; 3 Department of Brain and Behavioral Sciences, University of Pavia, 27100 Pavia, Italy; umberto.provenzani@unipv.it (U.P.); stefano.damiani@unipv.it (S.D.); natascia.brondino@unipv.it (N.B.); 4 Department of Biostatistics and Health Informatics, Institute of Psychiatry, Psychology & Neuroscience, King’s College London, London SE5 8AB, UK; 5 Department of Psychiatry, University of Oxford, Oxford OX3 7JX, UK; philip.mcguire@psych.ox.ac.uk; 6 NIHR Oxford Health Biomedical Research Centre, Oxford OX3 7JX, UK; 7 OPEN Early Detection Service, Oxford Health NHS Foundation Trust, Oxford OX3 7JX, UK; 8 Department of Psychiatry and Psychotherapy, Ludwig-Maximilian-University Munich, 80336 Munich, Germany

**Keywords:** physical health, psychosis, risk, CHR-P

## Abstract

Background: The clinical high risk for psychosis (CHR-P) construct represents an opportunity for prevention and early intervention in young adults, but the relationship between risk for psychosis and physical health in these patients remains unclear. Methods: We conducted a RECORD-compliant clinical register-based cohort study, selecting the long-term cumulative risk of developing a persistent psychotic disorder as the primary outcome. We investigated associations between primary outcome and physical health data with Electronic Health Records at the South London and Maudsley (SLaM) NHS Trust, UK (January 2013–October 2020). We performed survival analyses using Kaplan-Meier curves, log-rank tests, and Cox proportional hazard models. Results: The database included 137 CHR-P subjects; 21 CHR-P developed psychosis during follow-up, and the cumulative incidence of psychosis risk was 4.9% at 1 year and 56.3% at 7 years. Log-rank tests suggested that psychosis risk might change between different levels of nicotine and alcohol dependence. Kaplan-Meier curve analyses indicated that non-hazardous drinkers may have a lower psychosis risk than non-drinkers. In the Cox proportional hazard model, nicotine dependence presented a hazard ratio of 1.34 (95% CI: 1.1–1.64) (*p* = 0.01), indicating a 34% increase in psychosis risk for every additional point on the Fagerström Test for Nicotine Dependence. Conclusions: Our findings suggest that a comprehensive assessment of tobacco and alcohol use, diet, and physical activity in CHR-P subjects is key to understanding how physical health contributes to psychosis risk.

## 1. Introduction

The CHR-P—Clinical High Risk for Psychosis—populations [1,2] present a substantially higher risk of transitioning to a first episode of psychosis compared to the general population, recently estimated at around 25% within 3 years [3]. These subjects might develop psychosis up to 10 years after the initial presentation [4], with longer-term longitudinal studies finding a 38% transition rate at 16 years [5].

Most of these patients will develop an ICD/DSM schizophrenia-spectrum disorder [6], but there is more uncertainty around non-transitioned CHR-P subjects as a high percentage do not reach clinical and functional recovery and present at least one mental disorder at long-term follow-ups [7]. Indeed, aside from the classical 3 CHR-P subgroups—Attenuated Psychotic Symptoms (APS), Brief Limited Intermitted Psychotic Symptoms (BLIPS), and Genetic Risk and Deterioration Syndrome (GRD)—that define the construct [8], CHR-P individuals may present comorbidities such as affective or anxiety disorders [9]. 

Impaired global functioning is a key component of the clinical construct [10] and might represent a predictor of transition to psychosis [11,12]. Amongst the other psychosis risk factors in CHR-P subjects, physical health data are still under-investigated. For example, in one of the most recent meta-analyses on the topic [13], only 3 out of 44 studies investigated physical conditions, whilst more attention was given, for instance, to substance use. 

Despite these gaps in the literature and the fact that current CHR-P assessments are entirely based on psychopathological features [8,14], the CHR-P phase represents a window of opportunity for prevention and early intervention in young cohorts (14–35 years), including the opportunity to ameliorate crucial physical ill-health trajectories [15]. This opportunity is particularly relevant in view of the alarming weight of serious mental illnesses on the overall disease burden worldwide, more precisely the 21.2% of total years lived with disabilities [16]. In terms of physical health, subjects affected by schizophrenia have a life expectancy reduced by approximately 10 to 30 years compared to the general population [17,18,19] and preventable cardiovascular risk factors such as tobacco use, abdominal obesity, a sedentary lifestyle, and a diet with high levels of saturated fats play a main role in this discrepancy [20,21].

Interestingly, some CHR-P subjects also present a higher prevalence of cardiometabolic risk factors compared to age-matched controls (e.g., increased blood pressure, waist circumference, and fasting blood glucose) [22]. This vulnerability has also been associated with modifiable physical health behaviours in CHR-P, such as reduced physical activity and increased rates of smoking and alcohol abuse [23,24,25,26,27].

There are several reasons to promote good physical health and lifestyles during the CHR-P phase. As discussed above, a large proportion of CHR-P subjects develop comorbid disorders (e.g., mood, anxiety) at various stages [9], which are also correlated with physical health deterioration [28]. Second, in CHR-P individuals who will transition to psychosis, adopting a preventative approach in the earliest stage is associated with better long-term outcomes [29], as psychosis is frequently associated with a wide range of comorbid and multiple physical health illnesses [30] and often progresses to chronic, severe conditions [31].

Finally, several physical health outcomes, such as tobacco use [32,33], substance use, including alcohol [34], low levels of physical activity [35], and dietary components such as omega-3 fatty acids [36], have been proposed as risk factors for psychosis, and thus physical health interventions might reduce the risk of transitioning to psychosis for CHR-P subjects. However, only a limited number of studies [37,38] investigated the degree of causality in these relationships. 

Even though promoting physical health in these clinical populations is likely to be beneficial [39], physical health outcomes are often not monitored in CHR-P services [40], a problem shared with psychiatric services more broadly [41]. High-profile research focused on physical health is still scarce [40], but recently it was found that well-tolerated exercise in the CHR-P phase might improve fitness, cognitive performance, and the severity of attenuated positive symptoms [42]. Attention to the physical health of patients suffering from mental disorders has been increasing in the last few years. For instance, one of the pillars of the “NHS five year forward view” [43] focused on increasing physical health checks in these clinical populations. However, more robust research evidence is required to help bridge the gap between scientific understanding and clinical need and practice. More comprehensive and precise data would offer a better-informed view of feasible physical health interventions for these patients [44] and an understanding of the significance of these outcomes in CHR-P patients who develop a first episode of psychosis.

### Aim of the Study

The primary aim of this study was to illustrate the relationship between the risk of transition to psychosis in a cohort of CHR-P service users and physical health data, routinely collected via Electronic Health Records (EHR) and through validated questionnaires. We hypothesised that CHR-P subjects with poorer physical health and lifestyle (high nicotine or alcohol dependency, low level of physical activity, or unbalanced diet) have an increased risk of transitioning to psychosis, in line with previous findings of studies focusing on physical outcomes in psychosis [33,45]. Potential findings may shed light on the role of physical health outcomes in the genesis of psychosis and call for more preventative interventions tailored to the physical health needs of these patients.

## 2. Materials and Methods

### 2.1. Design

A clinical cohort study using Electronic Health Records (EHR) conducted in accordance with the REporting of studies Conducted using Observational Routinely collected health Data (RECORD) Statement [46].

### 2.2. Data Source

EHR data on routine physical health checks [47] from all individuals from January 2013 until October 2020, managed by the South London and Maudsley (SLaM) National Health Service (NHS) Foundation Trust, UK. The data source EHR employed in the current study provides contemporaneous EHR and ‘real-world’ data on routine mental healthcare from all patients managed by SLaM. SLaM is a UK NHS mental health trust that provides secondary mental health care to a population of 1.36 million individuals in South London (Lambeth, Southwark, Lewisham, and Croydon boroughs), with around 545,000 subjects aged 16–35. In SLaM, there is one of the highest rates of psychosis in the world [48]. In terms of the quality of SLaM/CRIS records, SLaM was an early pioneer of EHR, and the Trust is effectively digitised and paper-free. SLaM has a near-monopoly in terms of secondary mental healthcare provision in its local catchment area, and it is a legal requirement for SLaM healthcare professionals to keep these records up-to-date [49]. Whereas many national registers capture only those patients who have been hospitalised, the SLaM EHR register contains the full clinical records of all patients, which are continually updated throughout their care, regardless of discharges from and/or referrals to other services. 

### 2.3. Study Population

OASIS (Outreach and Support in South London) was set up in 2001, and it is one of the oldest early detection CHR-P services in the UK [50,51]. The service is focused on the identification, prognostic assessment, treatment (pharmacological, psychological, and psychoeducational), and clinical follow-up of help-seeking CHR-P individuals aged 14–35 years, serving the SLaM catchment area. OASIS is integrated into the Pan-London Network for Psychosis-prevention (PNP) [52]. The study population included a sample of all individuals accessing OASIS in the period from January 2013 to October 2020, assessed with the Comprehensive Assessment of At-Risk Mental State (CAARMS) [8] and meeting CHR-P criteria: Attenuated Psychotic Symptoms (APS), Brief Limited Intermittent Psychotic Symptoms (BLIPS), and Genetic Risk and Deterioration Syndrome (GRD). All OASIS staff undergo extensive psychometric training to ensure high reliability in the designation of at-risk cases [53]. The OASIS population can be considered representative of the general CHR-P sample since the level of risk enrichment observed (pretest risk [54]: 14.6% at more than 3 years [55]) aligns with that observed in CHR-P services worldwide (meta-analytical pretest risk: 15% at more than 3 years [56]). 

### 2.4. Assessment Instruments

Baseline assessment of CHR-P subjects includes a routine and comprehensive medical examination for physical parameters (e.g., BMI, heart rate, systolic pressure; see “*Study measures*”), which is complemented by the following validated questionnaires, in line with NICE (National Institute for Health and Care Excellence) Clinical Guideline 178 [57].

1. The Fagerström Test for Nicotine Dependence (FTND) [58] is a standardised instrument consisting of six questions exploring daily cigarette consumption, compulsive use, and dependence. The score ranges from 0 to 10 (with higher scores indicating a most severe level of dependence on nicotine). More precisely, scores from 0 to 2 indicate a low level of dependence, from 3 to 4 low-moderate dependence, from 5 to 7 moderate dependence, and more than 8 a high level of dependence. For people that use other types of nicotine consumption other than cigarette smoking (e.g., e-cigarette, nicotine gum, or nicotine patches), we have investigated habits and reported information in adapted versions of FTND already used in previous literature (for instance, the equivalence of 10 vape nicotine puffs for a cigarette [59] or a re-worded test for gum users [60]).
2. AUDIT (Alcohol Use Disorder Identification Test) [61] consists of 10 self-administered questions to investigate alcohol use disorder. When the AUDIT-C score, which includes core questions regarding alcohol units consumed and frequency of drinking, is equal to or above 5, it might indicate hazardous drinking. Regarding the AUDIT total score, a low level of risk is identified with an overall score between 0 and 7, and the range from 8 to 15 is the most appropriate for simple advice focused on the reduction of drinking. Higher scores (up to 19) suggest a need for brief counselling and continuous monitoring, while a complete diagnostic evaluation for alcoholic dependence is warranted for scores over 20.
3. DINE (Dietary Instrument for Nutritional Education) [62] is a structured interview investigating dietary fibre and fat (unsaturated and saturated) intake. Scores for fibres and fats are rated into three different categories: low (under 30), medium (between 30 and 40), and high (more than 40). Scores for unsaturated fats are rated as low (less than 6), medium (6–9) and high (more than 9).
4. IPAQ (International Physical Health Questionnaire) [63] rates the level of physical activity. This tool comprises three different categories of physical activity based on intensity (vigorous, moderate, and walking) and quantifies the amount of time spent sitting. Scores can also be expressed as a continuous variable with METs (estimating resting energy expenditure) [64].

### 2.5. Study Measures

The primary outcome was the long-term (up to 7 years) cumulative incidence (risk) of developing a persistent psychotic disorder, defined as the onset of the first ICD-10 diagnosis of a non-organic psychotic disorder (Table S1) from a CHR-P stage and association of the outcome with the physical health data. The start of the follow-up period was defined as the time of acceptance into the secondary mental health service (OASIS), and the time of an event was defined as the transition to psychosis. The patients’ time lost to follow-up was used for censoring and indicated by the last clinical entry to the EHR. Baseline outcome variables included were:

1. Sociodemographic parameters: age, sex, ethnicity.
2. Physical health data:
  o Tobacco use: tobacco smoker status (yes/no), number of daily cigarettes, FTND score.
  o Alcohol use: alcohol drinker status (yes/no), AUDIT-C, and AUDIT total score.
  o Type of diet: DINE fibre score, DINE saturated fat score, DINE unsaturated fat score.
  o Physical activity: IPAQ vigorous, moderate, and walking activity (minutes per week), IPAQ time spent sitting (minutes per week), MET levels (continuous variable).
  o Physical parameters: BMI (body mass index), waist circumference in centimetres, heart rate in beats per minute (bpm), respiratory rate in acts per minute (apm), systolic and diastolic pressure in mmHg.

### 2.6. Statistical Analysis

This clinical register-based cohort study was conducted according to the REporting of studies Conducted using Observational Routinely collected health Data (RECORD) Statement [46] (Table S2). Sociodemographic and physical health data of the sample (including missing data) were described with mean and standard deviation (SD) for continuous variables, and absolute and relative frequencies were used for categorical variables stratified by transition to psychosis.

As previously mentioned, the primary aim of the analysis was to investigate the association between physical health data and the risk of transitioning to psychosis. Firstly, the physical health data were categorised into strata to explore differences between groups through well-established survival analysis methods, by visually examining Kaplan-Maier survival curves [65], and by the formal assessment of between-group differences through the results of log-rank tests [66]. The categorisation of variables was structured as follows: (i) smoking: smokers vs. non-smokers; (ii) nicotine dependence: non-smokers—0 FTND score, low dependence < 4 FTND score, moderate dependence < 7, and high dependence > 7 FTND score; (iii) drinking status: drinkers vs. non-drinkers; (iv) alcohol use: non-drinkers—0 AUDIT score, non-hazardous drinkers < 8 AUDIT score, and hazardous drinkers ≥ 8 AUDIT score; (v) physical activity: vigorous, moderate, and walking activity were transformed into MET scores so that patients with MET scores less than 3000 were assigned to the inactive group and those with MET scores larger than 3000 to the active group; (vi) for DINE questionnaires, we used the categories illustrated in the “*Assessment Instruments*” section for fibre, saturated fat, and unsaturated fat subsets. 

The second part of the analysis consisted of quantifying the significance and magnitude of the association between the physical health data and psychosis risk with the Cox proportional hazard model, using recorded time to psychosis and censoring data [67]. Four measures were selected to be investigated by the Cox model. These included the FTND score as a measure of nicotine dependence, the AUDIT score as a measure of alcohol use, the fibre score in the DINE interview, and the MET score as a measure of physical activity. The four measures were selected as they were believed to capture the most information by their continuous nature, indicating the intensity of each physical health data, focusing on some modifiable risk factors, and on measures collected in a more rigorous way. Four Cox proportional hazard models were run with each of the four measures adjusted by the basic confounders of age, gender, and ethnicity. The four Cox models were inspected for influential observations by examining the standardised DFBETA values. Observations exceeding the 0.2 DFBETA threshold were excluded in the sensitivity analysis [68]. To adjust for multiple comparisons, the Benjamini-Hochberg correction was used [69]. 

All analyses were conducted in R, version 4.2.3 [70], using the ‘survival’ package.

## 3. Results

### 3.1. Sample Characteristics

The final database included 137 CHR-P subjects, 57 (41%) females and 80 (59%) males. The mean age was 23.65 ± 5.38 years (range from 14 to 36). The majority of the sample comprised White (39%) and Black British (21%) subjects. In terms of physical health outcomes, 40% of CHR-P subjects smoked tobacco, 77% drank alcohol, 63% had low fibre intake, and 72% were physically inactive (MET score of less than 3000). The mean follow-up time was 806 ± 634 days (range from 20 to 2785) (Table 1). The clinical characteristics and physical parameters of the full cohort were described elsewhere [24].

**Figure 1:**
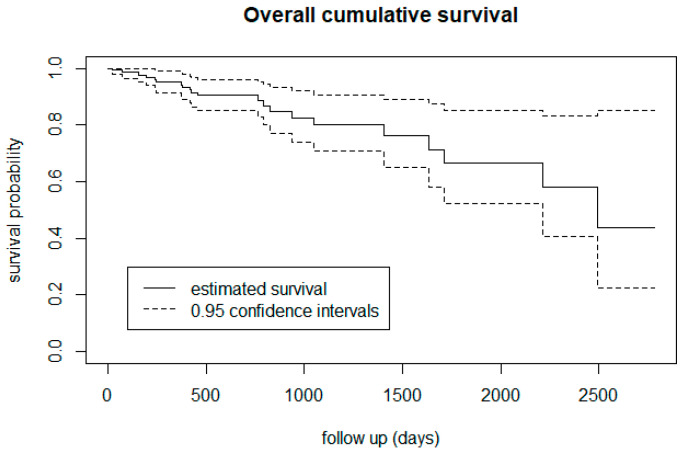
Cumulative incidence of risk of developing psychosis in the OASIS CHR-P sample across the follow-up. The decreasing trajectory is an expression of cumulative transitions to psychosis.

We observed 21 (15%) events (transitions to psychotic disorders) during the study period, 9 (16%) among females and 12 among males (15%) CHR-P individuals. The mean time to transition to psychotic disorders was 2098 days (95% CI: 1847–2349). The cumulative incidence (Kaplan-Meier survival function) of risk of developing psychotic disorders was 4.9% at 1 year (95% CI: 1.2–8.6%), 9.6% at 2 years (95% CI: 4.1–15.1%), 19.9% at 3 years (95% CI: 10.1–29.7%), 23.9% at 4 years (95% CI: 11.8–36.1%), 33.3% at 5 and 6 years (95% CI: 17.0–49.6%), and 56.3% at 7 years (95% CI: 27.1–85.5%) (Figure 1).

### 3.2. Physical Health Data and Transition in the CHR-P Sample

#### 3.2.1. Tobacco Use

**Figure 2:**
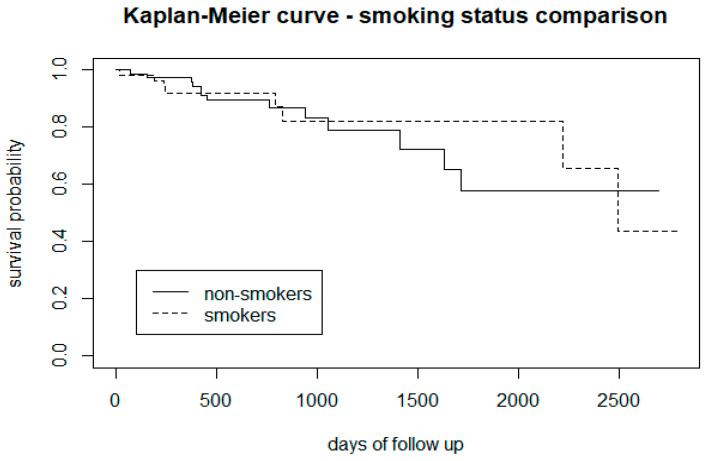
Representation of CHR-P smokers vs. non-smokers (scoring 0 at FTND).

**Figure 3:**
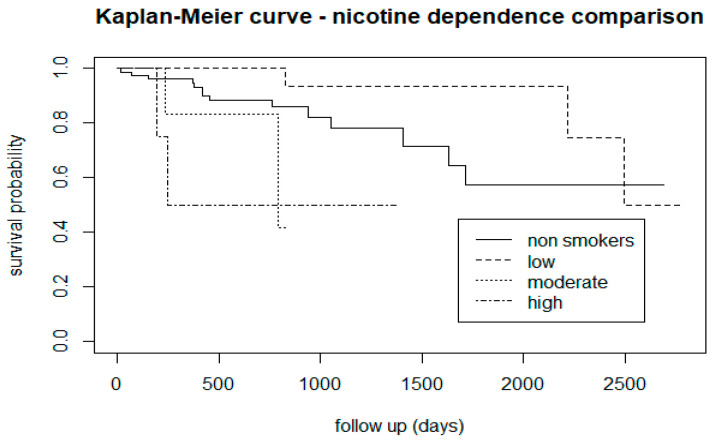
Comparison between CHR-P groups at different levels of nicotine dependence.

The comparison between smoker and non-smoker CHR-P subjects (the latter scoring 0 on the FTND) showed that smoking tobacco is associated with a lower risk of transition, especially after 1000 days of follow-up (Figure 2). Considering the different levels of nicotine dependence as per the FTND, relative to non-smokers, it appears that patients with a moderate to high level of tobacco dependence have a higher risk of developing psychosis, and people with a low level of dependence are less prone to developing psychosis (Figure 3).

#### 3.2.2. Alcohol Use

**Figure 4:**
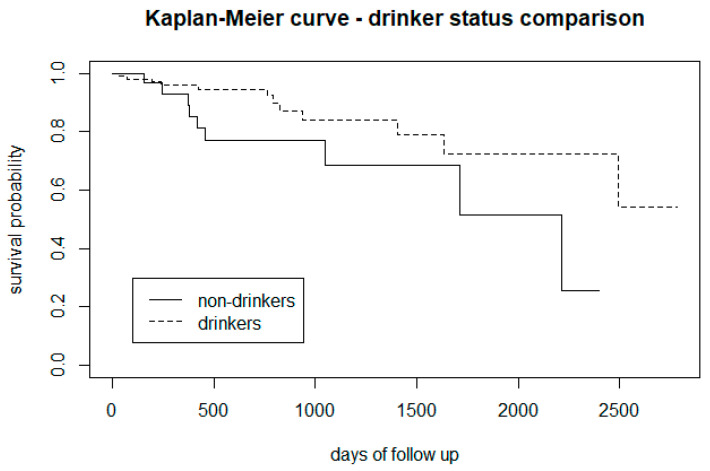
Risk of transition to psychosis between CHR-P individuals that drink alcohol vs. non-drinkers.

**Figure 5:**
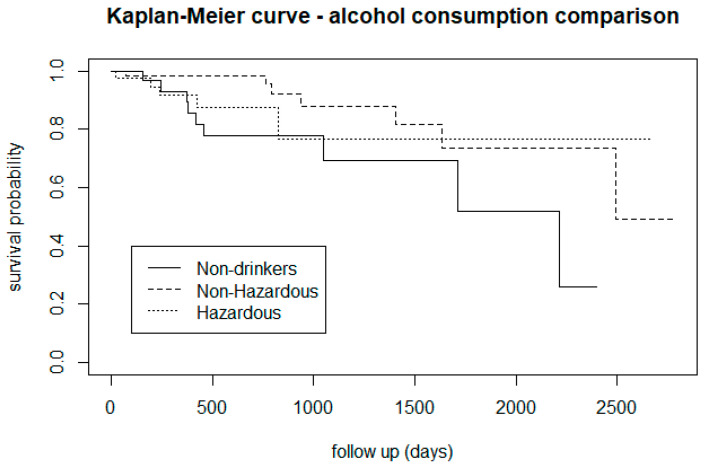
Risk of transition to psychosis in CHR-P subjects, stratified by level of alcohol dependency.

Figure 4 indicated that for almost the entire follow-up period, CHR-P subjects who drink alcohol were less at risk of developing psychosis than those who do not drink. However, Figure 5 (Kaplan-Maier graph stratified by distinct levels of alcohol dependence) indicated that non-hazardous drinkers may have a lower risk of transition than non-drinkers.

#### 3.2.3. Type of Diet

**Figure 6:**
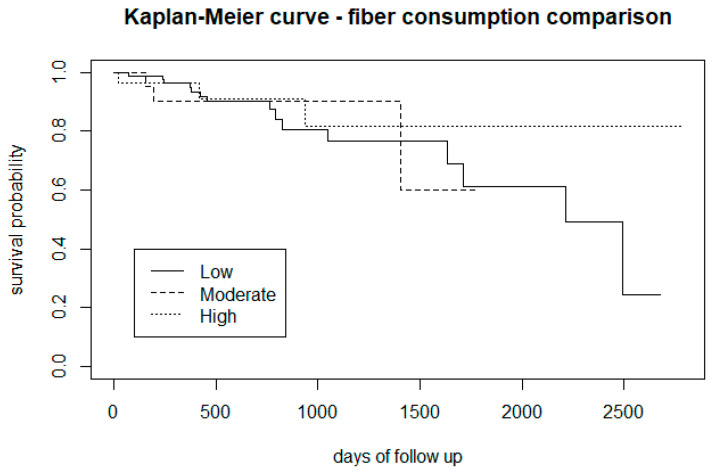
Comparison of the risk of transition to psychosis between CHR-P subjects with a high, moderate, and low fibre intake.

A visual examination of the Kaplan-Meier curve for fibre intake (Figure 6) showed that CHR-P subjects who self-report high fibre intake present with a lower risk of transitioning to psychosis. Graphs related to saturated and unsaturated fat are appended in Supplementary Materials (Figures S1 and S2).

#### 3.2.4. Physical Activity

**Figure 7:**
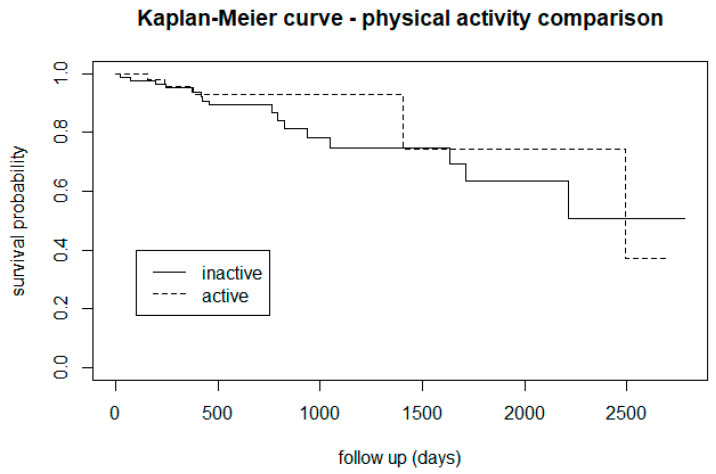
Psychosis risk comparison between physically active CHR-P subjects (>3000 METs) and inactive subjects (<3000 METs).

Visually exploring the Kaplan-Meier graphs suggested that being physically active may be a protective factor against the transition to psychosis (Figure 7).

#### 3.2.5. Physical Parameters

**Figure 8:**
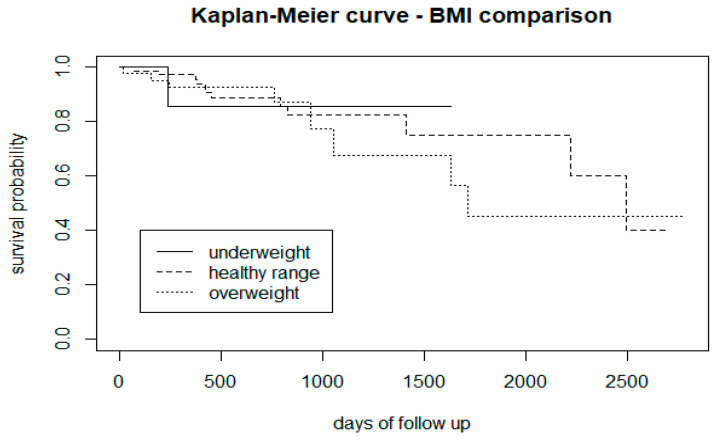
Stratification of psychosis risk in CHR-P subjects with normal, low, and high BMI.

After 1000 days of follow-up, the Kaplan-Meier curve suggested that overweight CHR-P subjects are more at risk of transitioning to psychosis (Figure 8).

### 3.3. Log-Rank Tests

The log-rank tests indicated that there may be significant differences between different nicotine dependence groups and psychosis risk. Further exploration of differences between individual nicotine strata revealed that significant differences exist when the low and moderate-high groups are compared, as well as when non-smokers are compared with moderate-high groups (Table 2). The evidence for the difference between various alcohol consumption groups and transition risk is less clear, with a χ^2^ of 6 and a corresponding *p*-value of 0.05. The results of the log-rank tests for fibre intake, physical activity levels, and BMI values did not indicate significant differences between groups.

### 3.4. Cox Proportional Hazard Model 

The Cox proportional hazard model results are presented in Table 3. Nicotine dependence as measured by the FTND resulted in a Hazard Ratio of 1.34 (95% CI: 1.1–1.64) with an adjusted Benjamini-Hochberg correction *p*-value of 0.01, which suggests a 34% increase in psychosis risk with every additional point in the FTND score. The confidence intervals of the hazard ratios for the AUDIT score, DINE (fibre score), and the MET physical activity score did not indicate significant associations between these three measures and the transition to psychosis. The DFBETA analysis did not find any influential observations related to the physical health measures at the 0.2 threshold. The only influential observations (DFBETA > 0.2) were found for ethnicity. In sensitivity analysis, after the exclusion of the influential observations, the physical health coefficients changed only marginally, not influencing the interpretation.

## 4. Discussion

We conducted this study on one of the largest CHR-P cohorts (137 subjects) with a long-term follow-up, focusing on physical health data and psychosis risk. This is a subset of a larger dataset we used to describe CHR-P physical health in a cross-sectional design [24]. In the present study, 21 patients transitioned to psychosis across the follow-up period, with a cumulative psychosis risk of 56.3% at 7 years.

From the survival analysis, people who smoke tobacco presented a lower proportion of transition to psychosis (Figure 2), but stratifying the level of nicotine dependence, we noticed that subjects with a moderate to high dependence have a higher risk of psychosis, while people with a low tobacco dependence presented a lower risk than non-smokers (Figure 3). More precisely, we found that smokers with low-dependence are much less at risk than people with moderate-high dependence (Table 2). Kaplan-Meier curves of alcohol status and alcohol dependence showed that patients who are abstinent from alcohol are more prone to developing psychosis (Figure 4). In terms of diet, we found that a low fibre intake might be related to a higher risk of psychosis (Figure 6). In this cohort, higher levels of physical activity seemed to be a protective factor from psychosis (Figure 7), and subjects with a high BMI might have a higher risk after 1000 days of follow-up, but initially underweight is more related to psychosis risk (Figure 8). These results should be interpreted with caution, as log-rank tests showed a significant difference between distinct levels of dependence on nicotine and alcohol and psychosis risk (Table 2), and the Cox proportional hazard model showed an increment of 34% of psychosis per point only for the FTND (nicotine dependence) score (Table 3). 

These latter findings corroborate evidence of the correlation between tobacco use in CHR-P individuals and psychosis. This is in line with the formulation of tobacco smoking as a risk factor [23,32,33], one of the current hypotheses, along with self-medication [71]. Unfortunately, we were not able to control for confounders such as cannabis use, especially high-potency strains, which are also strongly associated with the onset of first-episode psychosis [72,73] and frequently used by subjects that smoke tobacco [74]. Despite the high prevalence of tobacco smoking in CHR-P subjects [23,24], only a few longitudinal studies have investigated the association between tobacco use and transition to psychosis from a CHR-P state [75,76,77,78], and our findings require corroboration from future studies that investigate the association in terms of causality. More studies are also needed to investigate the relevant difference in psychosis risk between low and moderate-high nicotine dependence, as it might involve additional confounders, such as potential social factors. 

In terms of alcohol use, we replicated the finding of Buchy and colleagues [37,79] who found that low levels of alcohol use were associated with a higher risk of transitioning to psychosis. Again, the result needs to be interpreted with caution, as even though alcohol intake rates are high in CHR-P subjects [24,38], there is a relatively low prevalence of cases of alcohol abuse/high dependence. This may reflect the fact that this subgroup of patients is primarily treated by addiction services, and this might affect the ability to detect the effect of alcohol on psychosis development. We also need to consider the possibility that low alcohol use might be interpreted as a proxy measure for social functioning [80]. Indeed, in the survival analysis, subjects with low alcohol dependence appeared less at risk of transition than abstinent subjects. These findings were also supported by the result of the log-rank test, which found a significant difference in transition risk across different levels of alcohol dependence. Overall, the role of alcohol in the development of psychosis is still not completely clear, as it does not predict the transition to psychosis in different studies [37,79], but it was detected as an important confounder between cannabis use and psychosis conversion in a high-risk sample [81]. These observations underline the importance of developing tailored alcohol intake monitoring and related preventative interventions in the CHR-P phase.

We made one of the first attempts to assess the dietary intake of a CHR-P cohort. Diet has been identified as a modifiable risk factor in depression, and higher dietary intake of energy, sodium [82], refined carbohydrates, and total fats, as well as a lower intake of fibre and omega-3 and omega-6 fatty acids (FA), are related to the psychosis spectrum, but specific dietary guidelines are still not available [83]. This dimension has not been systematically evaluated yet in subjects at risk of developing psychosis. There is evidence [84] that CHR-P subjects report an increased intake of calories and saturated FA and reduced protein consumption compared to healthy controls. Moreover, a prospective study [85] found that CHR-Ps consumed significantly more calories than controls. More recently, a study showed a relatively low red blood cell omega-3 index in CHR-P subjects [86], and cross-sectional data found a positive correlation between intake of omega-3 FA and functional status [86]. In terms of prediction of functional outcomes, results are less clear, but combined concentrations of baseline erythrocyte membrane FA have been found to predict functional enhancement in CHR-P subjects [87]. In one Polish study [36], CHR-P subjects who transitioned to psychosis reported consuming less omega-3 FA than the non-converters. In terms of interventions, a multicentre RCT [88] failed to replicate the result of a single-centre study [89,90], showing that omega-3 polyunsaturated FAs are not effective in preventing transition to psychosis when evidence-based psychological interventions are available. However, a longitudinal analysis of biomarkers showed that an increase in the omega-3 index predicted better symptomatology and functional outcomes [91]. 

Given the lack of validated dietary assessment tools for patients suffering from serious mental illnesses [92], the DINE questionnaire was challenging to administer, especially in saturated and unsaturated fat sections (e.g., items with a high weight in the total score were particularly difficult to assess). This calls for the implementation of dietary assessment tools tailored for clinical populations suffering from mental disorders [92,93]. The fibre section of the DINE questionnaire was easier to administer, and the results appeared to be more reliable. This is noteworthy, as in our cross-sectional study, 60% of CHR-P subjects presented with a low fibre intake [24], in line with psychosis patients [83]. In our survival analysis, people with a lower fibre intake appeared to transition more than people with a higher proportion of fibre in their diet, but this result was not confirmed by the log-rank test nor the Cox proportional hazard model. In a recent review, Teasdale and colleagues [82] recommended further research into dietary intake in the pre-illness onset phase to understand “whether any dietary factors may indicate the onset of the illness”.

People with psychosis present with reduced physical activity levels [94,95,96] and spend more time in sedentary behaviour [95] than the general population, which may contribute to their increased cardiometabolic risk [97,98,99]. CHR-P populations also present with reduced levels of physical activity [25], and our cross-sectional study [24] found that in CHR-P subjects, averages of physical activity levels were far below UK national guidelines [100].

A recent meta-analysis found that people with high levels of self-reported physical activity had reduced odds of developing psychosis, but the association was no longer significant when adjusted for covariates [101]. The literature on the topic is still scarce, but one Finnish cohort study identified low levels of physical activity in childhood/early adolescence as an independent predictor of psychosis [35]. Similarly, a birth cohort study found that subjects who later transitioned to psychosis were more inactive during their adolescence [102], and adolescents with parents with psychosis who were more engaged in physical activity were 24% less likely to develop psychosis [103]. This is in line with our survival analysis that showed that physically active CHR-P individuals (IPAQ-MET score) are less likely to develop psychosis, but our result was not confirmed using the log-rank test or the Cox proportional hazard model. The Lancet Psychiatry Commission [104] advocated physical activity as a core component for preventative interventions from the earliest stages of mental illnesses to protect physical health from illness onset and prevent physical health comorbidity from developing. A recent study [105] confirmed that CHR-P individuals are less fit than controls and that their self-report items did not reflect objective indices of fitness, perhaps reflecting elements of grandiosity or avolition. This adds further caution when interpreting results about physical activity that rely exclusively on self-reported measures. Given the effect of physical exercise on brain plasticity [106] and potentially also in CHR-P subjects [42,107], it is plausible that it may help to protect CHR-P against psychosis beyond the improvement of their fitness, global functioning, attenuated symptoms, and cognitive performance [42,107]. There is evidence that service users find interventions on physical activity feasible and acceptable [108]. 

In terms of physical parameters, our cross-sectional study [24] found that CHR-P physical measures were in line with the matched UK general population, similar to a previous review [25], but in contrast with the NAPLS study, which found high rates of cardiometabolic abnormalities, including obesity [86]. In the current study, CHR-P individuals with high BMI appeared more at risk of developing psychosis after 1000 days of follow-up in the survival analysis, but this result should be interpreted with caution because of the low number of transitions in the later stages of follow-up.

Results from a Finnish birth cohort [109] showed that being underweight but not overweight in adolescence independently predicts the later development of non-affective psychosis. Classically, low birthweight has been interpreted as a risk factor for schizophrenia [110], and several studies have reported associations between non-affective psychosis and low BMI during childhood and adolescence/young adulthood [111]. The literature presents different associations between BMI and different clinical features in the CHR-P phase. For instance, in medicated CHR-P subjects (with antidepressants/antipsychotics) BMI was negatively correlated with positive symptoms [112]. An increase in BMI was associated with baseline molecular circulating lipids [113]. BMI was also positively correlated with leptin levels [114] and was negatively associated with polyunsaturated FA levels [115]. Moreover, in subjects with a familial risk of psychosis, a higher BMI was related to white matter abnormalities [116]. 

Only a few studies have reported waist circumference values for CHR-P individuals [22,84,114,117]. 

Overall, BMI and the other anthropometric measures are still not well characterised in CHR-P subjects [105], in line with various other potential risk factors for psychosis. For instance, high systolic blood pressure was found to be weakly correlated to an increased risk of schizophrenia in a recent GWAS study [118]. However, a single study found systolic pressure to be significantly lower in CHR-P individuals than in controls during a stress test [117]. Higher blood pressure and resting heart rate (RHR) values in adolescence predicted schizophrenia in adulthood in male subjects from a large cohort study [119]. Heart rate (HR) is considered an expression of autonomic functioning [120,121], which is altered in many psychiatric syndromes [122,123]. In the CHR-P phase, findings are contradictory: Clamor and colleagues [124] did not find any difference in heart parameters compared to healthy controls, while Counotte et al. [125] reported associations between psychosis liability and increased HR and HR variability (HRV). Furthermore, Kocsis [126] found an increased RHR in CHR-P patients relative to healthy controls, and increased RHR was positively correlated with CAARMS severity and distress scores. Low HRV was instead correlated with antipsychotic/antidepressant use in a longitudinal study [127] in CHR-P individuals.

In the end, only a small proportion of studies systematically report the physical measures of CHR-P cohorts [25], especially in relation to the transition to psychosis. This calls for further high-quality research to facilitate a more comprehensive understanding of the role of physical measures in the context of psychosis onset. 

In terms of limitations, we did not assess physical health data at different time points, and so we cannot address potential changes in these habits across the follow-up period. In addition, we mainly used self-report measures, which might be susceptible to recall bias. The use of biomarkers would help to overcome this limitation. As mentioned, due to the lack of validated instruments to assess dietary intake for populations with serious mental illness, the administration of the DINE questionnaire was particularly challenging. We did not control for substance use (cannabis use may be of particular importance), so our findings around tobacco and the transition to psychosis should be interpreted with caution, given the frequent cases of polysubstance abuse. Finally, data on education were not available, and we did not control for religion, spirituality, and family bonds [128], which might also represent potential confounders given their potential influence on alcohol and dietary habits or their reported effects on treatment maintenance, sociality, and health practices [129,130].

## 5. Conclusions

This study investigated the relationship between different physical health data and the transition to psychosis in a large cohort of CHR-P service users, finding that an increase in nicotine dependence is related to a substantial increase in psychosis risk, as confirmed by the Cox proportional hazard model. We also conducted survival analyses on alcohol use, dietary intake, levels of physical activity, and BMI, finding that non-drinkers were more likely to develop psychosis, as were CHR-P subjects with low fibre intake and low physical activity levels. In terms of anthropometric measures, a higher BMI was associated with greater psychosis risk after the first 1000 days. 

These findings call for monitoring data on physical health and lifestyle in CHR-P subjects to increase our understanding of their potential role in psychosis onset and to implement tailored interventions targeting unhealthy daily habits. Indeed, interventions aimed at reducing alcohol and tobacco use, promoting a balanced diet, and promoting physical activity in line with national guidelines would constitute favourable and generalisable treatments for CHR-P, with potential effectiveness in also improving mental health outcomes in this clinical population.

## Figures and Tables

**Table 1:** Sample description.

Patient Characteristics Stratified by Psychosis Transition		
	Non-Transitioned	Transitioned
*n*	116	21
**Gender**		
Male	68 (58.6)	12 (57.1)
Female	48 (41.4)	9 (42.9)
**Age Group**		
<20	34 (29.3)	3 (14.3)
20–25	38 (32.8)	10 (47.6)
26–30	24 (20.7)	4 (19.0)
>30	20 (17.2)	4 (19.0)
**Ethnicity**		
White	51 (44.0)	2 (9.5)
Asian	2 (1.7)	2 (9.5)
Black African	10 (8.6)	5 (23.8)
Black Caribbean	3 (2.6)	1 (4.8)
Black British	22 (19.0)	7 (33.3)
Other	28 (24.1)	4 (19.0)
**Smoker Status**		
Yes	47 (40.5)	8 (38.1)
No	69 (59.5)	13 (61.9)
**Nicotine Dependence**		
Non-Smokers	69 (59.5)	14 (66.7)
Low	40 (34.5)	3 (14.3)
Moderate	3 (2.6)	2 (9.5)
High	4 (3.4)	2 (9.5)
**Drinking Status**		
Yes	93 (80.2)	12 (57.1)
No	23 (19.8)	9 (42.9)
**Alcohol Consumption**		
Non-Drinkers	25 (21.6)	9 (42.9)
Non-Hazardous	57 (49.1)	7 (33.3)
Hazardous	34 (29.3)	5 (23.8)
**Fibre Consumption**		
Low	72 (62.1)	15 (71.4)
Moderate	20 (17.2)	3 (14.3)
High	24 (20.7)	3 (14.3)
**Physical Activity**		
Active	35 (30.2)	3 (14.3)
Inactive	81 (69.8)	18 (85.7)
**BMI Category**		
Underweight	7 (6.3)	1 (4.8)
Healthy Range	67 (60.4)	12 (57.1)
Overweight	37 (33.3)	8 (38.1)

**Table 2:** Comparison of differences between groups with a log-rank test.

Log-Rank Test Group Comparison			
Feature	χ^2^	Degrees of Freedom	*p*-Value
Nicotine dependence: low, moderate, high, and non-smokers	12.8	3	0.005
Nicotine dependence: non-smokers and low	3.6	1	0.06
Nicotine dependence: low and moderate or high	14.1	1	0.002
Nicotine dependence: non-smokers and moderate or high	4.7	1	0.03
Alcohol consumption: non-drinkers, non-hazardous, and hazardous	6	2	0.05
Fibre consumption: low, moderate, and high	1.3	2	0.5
Physical activity: active and non-active group	0.8	1	0.4
Body mass index (BMI)	0.3	2	2

**Table 3:** Hazard ratios obtained from four separate Cox proportional hazard models adjusted by age, gender, and ethnicity.

Cox Regression Results					
Feature	Hazard Ratios	Lower 0.95	Upper 0.95	*p*-Value	Adjusted *p*-Value
FTND	1.34	1.1	1.64	0.0034	0.01
AUDIT	1.04	0.95	1.143	0.34	0.45
DINE (fibre score)	0.99	0.96	1.01	0.7	0.7
Physical Act (MET)	0.99	0.96	1.01	0.22	0.44

## Data Availability

The authors give no permission to share raw data.

## Supplementary Materials

The following supporting information can be downloaded at: https://www.mdpi.com/article/10.3390/biomedicines12030523/s1, Table S1: ICD-10 Diagnoses for non-organic psychotic disorders; Table S2: The RECORD statement—checklist of items, extended from the STROBE statement, that should be reported in observational studies using routinely collected health data; Figure S1: Stratification of psychosis risk in CHR-P subjects with low, medium, and high saturated fat intake; Figure S2: Stratification of psychosis risk in CHR-P subjects with low, medium, and high unsaturated fat intake.
