# Supplementary material for: Physical Health and Transition to Psychosis in People at Clinical High Risk
Source: Biomedicines. 2024 Feb 26;12(3):523. doi: 10.3390/biomedicines12030523 (PMC10968449; doi:10.3390/biomedicines12030523)

Supplementary materials

Table S1: ICD-10 Diagnoses for non-organic psychotic disorders

Psychotic disorders	F20.0	Paranoid schizophrenia
	F20.1	Hebephrenic schizophrenia
	F20.2	Catatonic schizophrenia
	F20.3	Undifferentiated schizophrenia
	F20.4	Post-schizophrenic depression
	F20.5	Residual schizophrenia
	F20.6	Simple schizophrenia
	F20.8	Other schizophrenia
	F20.81	Schizophreniform disorder
	F20.9	Schizophrenia, unspecified
	F25.0	Schizoaffective disorder, manic type
	F25.1	Schizoaffective disorder, depressive type
	F25.2	Schizoaffective disorder, mixed type
	F25.8	Other schizoaffective disorders
	F25.9	Schizoaffective disorder, unspecified
	F22.0	Delusional disorder
	F22.8	Other persistent delusional disorders
	F22.9	Persistent delusional disorder, unspecified
	F24	Induced delusional disorder
	F28	Other nonorganic psychotic disorders
	F29	Unspecified nonorganic psychosis
	F30.2	Mania with psychotic symptoms
	F31.2	Bipolar affective disorder, current episode manic with psychotic symptoms
	F31.5	Bipolar affective disorder, current episode severe depression with psychotic symptoms
	F32.3	Severe depressive episode with psychotic symptoms
	F33.3	Recurrent depressive disorder, current episode severe with psychotic symptoms
	F53.1	Severe mental and behavioural disorders associated with the puerperium, not elsewhere classified (post-partum psychosis)

Table S2: The RECORD statement – checklist of items, extended from the STROBE statement, that should be reported in observational studies using routinely collected health data.

	Item No.	STROBE items	Location in manuscript where items are reported	RECORD items	Location in manuscript where items are reported
**Title and abstract**					
	1	(a) Indicate the study's design with a commonly used term in the title or the abstract (b) Provide in the abstract an informative and balanced summary of what was done and what was found		RECORD 1.1: The type of data used should be specified in the title or abstract. When possible, the name of the databases used should be included.<br><br>RECORD 1.2: If applicable, the geographic region and timeframe within which the study took place should be reported in the title or abstract.<br><br>RECORD 1.3: If linkage between databases was conducted for the study, this should be clearly stated in the title or abstract.	
**Introduction**					
Background rationale	2	Explain the scientific background and rationale for the investigation being reported			
Objectives	3	State specific objectives, including any prespecified hypotheses			
**Methods**					
Study Design	4	Present key elements of study design early in the paper			

Setting	5	Describe the setting, locations, and relevant dates, including periods of recruitment, exposure, follow-up, and data collection			
Participants	6	*Cross-sectional study* - Give the eligibility criteria, and the sources and methods of selection of participants		RECORD 6.1: The methods of study population selection (such as codes or algorithms used to identify subjects) should be listed in detail. If this is not possible, an explanation should be provided.<br><br>RECORD 6.2: Any validation studies of the codes or algorithms used to select the population should be referenced. If validation was conducted for this study and not published elsewhere, detailed methods and results should be provided.<br><br>RECORD 6.3: If the study involved linkage of databases, consider use of a flow diagram or other graphical display to demonstrate the data linkage process, including the number of individuals with linked data at each stage.	
Variables	7	Clearly define all outcomes, exposures, predictors, potential confounders, and effect modifiers. Give diagnostic criteria, if applicable.		RECORD 7.1: A complete list of codes and algorithms used to classify exposures, outcomes, confounders, and effect modifiers should be provided. If these cannot be reported, an explanation should be provided.	

Data sources/ measurement	8	For each variable of interest, give sources of data and details of methods of assessment (measurement).<br>Describe comparability of assessment methods if there is more than one group			
Bias	9	Describe any efforts to address potential sources of bias			
Study size	10	Explain how the study size was arrived at			
Quantitative variables	11	Explain how quantitative variables were handled in the analyses. If applicable, describe which groupings were chosen, and why			
Statistical methods	12	(a) Describe all statistical methods, including those used to control for confounding<br>(b) Describe any methods used to examine subgroups and interactions<br>(c) Explain how missing data were addressed<br>(d) *Cross-sectional study* - If applicable, describe analytical methods taking account of sampling strategy<br>(e) Describe any sensitivity analyses			
Data access and cleaning methods		..		RECORD 12.1: Authors should describe the extent to which the investigators had access to the	

				database population used to create the study population.<br><br>RECORD 12.2: Authors should provide information on the data cleaning methods used in the study.	
Linkage		..		RECORD 12.3: State whether the study included person-level, institutional-level, or other data linkage across two or more databases. The methods of linkage and methods of linkage quality evaluation should be provided.	
**Results**					
Participants	13	(a) Report the numbers of individuals at each stage of the study (*e.g.*, numbers potentially eligible, examined for eligibility, confirmed eligible, included in the study, completing follow-up, and analysed)<br>(b) Give reasons for non-participation at each stage.<br>(c) Consider use of a flow diagram		RECORD 13.1: Describe in detail the selection of the persons included in the study (*i.e.,* study population selection) including filtering based on data quality, data availability and linkage. The selection of included persons can be described in the text and/or by means of the study flow diagram.	
Descriptive data	14	(a) Give characteristics of study participants (*e.g.*, demographic, clinical, social) and information on exposures and potential confounders<br>(b) Indicate the number of participants with missing data for each variable of interest			

Outcome data	15	*Cross-sectional study* - Report numbers of outcome events or summary measures			
Main results	16	(a) Give unadjusted estimates and, if applicable, confounder-adjusted estimates and their precision (e.g., 95% confidence interval). Make clear which confounders were adjusted for and why they were included<br>(b) Report category boundaries when continuous variables were categorized<br>(c) If relevant, consider translating estimates of relative risk into absolute risk for a meaningful time period			
Other analyses	17	Report other analyses done—e.g., analyses of subgroups and interactions, and sensitivity analyses			
**Discussion**					
Key results	18	Summarise key results with reference to study objectives			
Limitations	19	Discuss limitations of the study, taking into account sources of potential bias or imprecision. Discuss both direction and magnitude of any potential bias		RECORD 19.1: Discuss the implications of using data that were not created or collected to answer the specific research question(s). Include discussion of misclassification bias, unmeasured confounding, missing data, and changing eligibility over time, as they pertain to the study being reported.	

Interpretation	20	Give a cautious overall interpretation of results considering objectives, limitations, multiplicity of analyses, results from similar studies, and other relevant evidence			
Generalisability	21	Discuss the generalisability (external validity) of the study results			
**Other Information**					
Funding	22	Give the source of funding and the role of the funders for the present study and, if applicable, for the original study on which the present article is based			
Accessibility of protocol, raw data, and programming code		..		RECORD 22.1: Authors should provide information on how to access any supplemental information such as the study protocol, raw data, or programming code.	

*Reference: Benchimol EI, Smeeth L, Guttmann A, Harron K, Moher D, Petersen I, Sørensen HT, von Elm E, Langan SM, the RECORD Working Committee. The REporting of studies Conducted using Observational Routinely-collected health Data (RECORD) Statement. *PLoS Medicine* 2015; in press.

*Checklist is protected under Creative Commons Attribution (CC BY) license.

Figure S1: Stratification of psychosis risk in CHR-P subjects with low, medium, and high saturated fat intake.

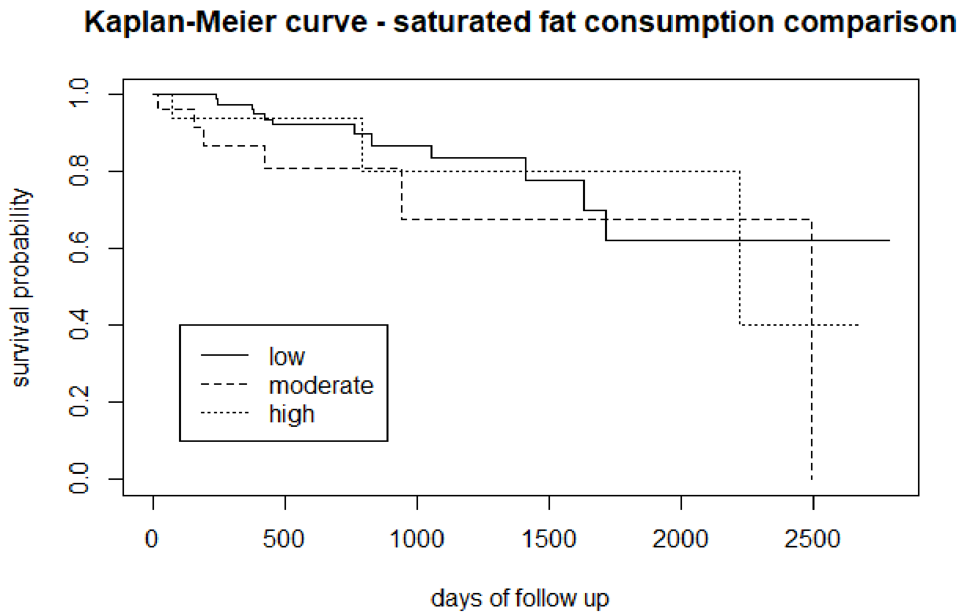

Figure S2: Stratification of psychosis risk in CHR-P subjects with low, medium, and high unsaturated fat intake.

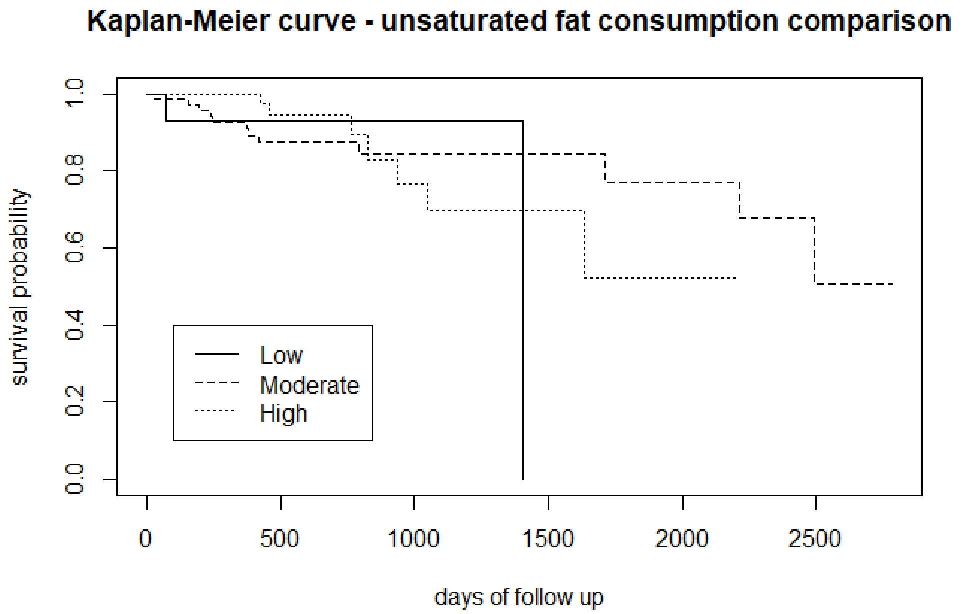

Supplement: Supplementary file 1 [file biomedicines-12-00523-s001.zip › biomedicines-2651506-supplementary.pdf]
